# First record of an agriolimacid slug in Southeast Asia – *Deroceras laeve* (O. F. Müller, 1774) (Gastropoda: Pulmonata) recently introduced to the Socialist Republic of Vietnam

**DOI:** 10.3897/BDJ.8.e59644

**Published:** 2020-12-03

**Authors:** Ivailo Kanev Dedov, Ulrich E. Schneppat, Heike Reise, Manh Quang Vu

**Affiliations:** 1 Institute of Biodiversity and Ecosystem Research, Bulgarian Academy of Sciences, Sofia 1113, 2 Gagarin Str., Bulgaria Institute of Biodiversity and Ecosystem Research, Bulgarian Academy of Sciences Sofia 1113, 2 Gagarin Str. Bulgaria; 2 retired, CH-7074, Churwalden-Malix, Sennereiweg 8, Switzerland retired CH-7074, Churwalden-Malix, Sennereiweg 8 Switzerland; 3 Senckenberg Museum of Natural History Görlitz, Am Museum 1, 02826 Görlitz, Germany Senckenberg Museum of Natural History Görlitz, Am Museum 1 02826 Görlitz Germany; 4 Hanoi National University of Education, Hanoi, 136 Xuan Thuy Rd., DHSP Cau Giay, Vietnam Hanoi National University of Education Hanoi, 136 Xuan Thuy Rd., DHSP Cau Giay Vietnam

**Keywords:** terrestrial slug, new introduction, COI, Gastropoda, Pulmonata, Agriolimacidae

## Abstract

Several individuals of the terrestrial slug *Deroceras laeve* were collected in 2018 in the Hoàng Liên Son mountain range of northern Vietnam. The three specimens which were investigated anatomically were aphallic or hemiphallic. A partial COI sequence verified the species identity. This is the first discovery of *D. laeve* and also of the slug family Agriolimacidae on the Indochinese Peninsula. The collecting site is situated near a cable-car station and below a tourist complex on Fansipan mountain, both of which had just been built by a Swiss-Austrian company between 2013 and 2016. This and the fact that the species had not been found elsewhere in the surrounding area, although searched for thoroughly, indicate that *D. laeve* is most probably a recent introduction, potentially with building material from Austria or Switzerland.

## Introduction

Globalisation of trade and long-distance travelling have led to unintended introductions of alien species worldwide, of which some have become serious pests causing problems in agriculture and horticulture or for human health (Cowie et al. 2009, Simberloff 2013). Their low mobility makes terrestrial molluscs usually poor dispersers, but some species have been very successful in hitchhiking on transports of goods (Cowie et al. 2009). Amongst terrestrial slugs, some Veronicellidae have been spread widely in the tropics and subtropics and become important agricultural pests. In the temperate regions of the world, a few European slug species of the genera *Deroceras* (Agriolimacidae), *Arion* (Arionidae) and *Ambigolimax* (Limacidae) have behaved similarly. *Deroceras reticulatum* (O. F. Müller, 1774), *Deroceras laeve* (O. F. Müller, 1774) and *Deroceras invadens* Reise, Hutchinson, Schunack & Schlitt, 2011 have spread not only across Europe but also to most other continents. These three species have also taken hold in the tropics (e.g. Welter-Schultes 2012, Hutchinson et al. 2014); it has been hypothesised that pest species from temperate regions are pre-adapted for the tropics, because the temperature fluctuations of their native area select for surviving high temperatures (Meyer and Cowie 2010). Particularly *D. laeve* inhabits an extremely wide range of habitats. Since Wiktor's (2000) monograph, in which he also stressed occurrences on tropical islands “such as New Guinea or others on the Pacific”, a number of further discoveries have turned up (see Discussion). However, there have been almost no records of any Agriolimacidae from SE Asia, potentially reflecting a lack of malacologists searching for or recognising them. We now report a small population of *D. laeve* in the high mountains of northern Vietnam, which is its first known occurrence on the Indochinese Peninsula.

## Material and methods

**Location**: Southeast Asia, Socialist Republic of Vietnam, Hoàng Liên Son Mountains range (the south-eastern extension of the Himalayan chain), Lao Cai Province, Municipality of Sa Pa, Hoàng Liên National Park, below Fansipan peak, northern-slope, 22.3054°N, 103.7751°E, 3020 m a.s.l.

**Figure 1.**
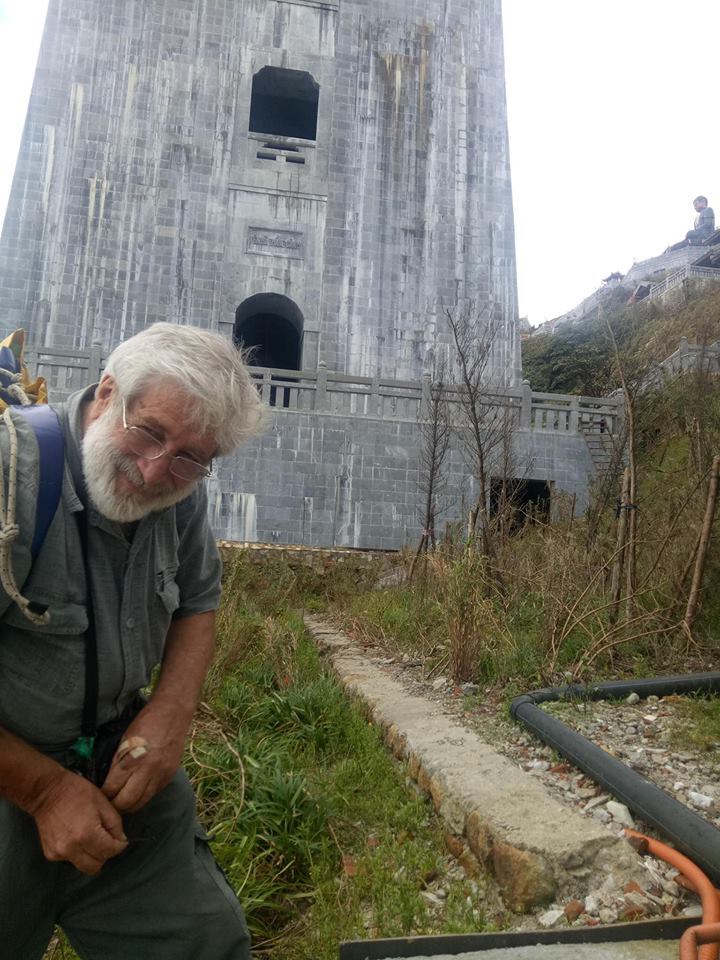
*Deroceras laeve*, Collection I. Dedov no. 40339/B, locality, Northern Vietnam, Lao Cai Province, Municipality of Sa Pa, below Fansipan peak

**Habitat**: Open, stony area with some sparse weedy vegetation, not far from the cable-car station and near temple buildings, surrounded by forest (Fig. 1). The top of Fansipan comprises disturbed habitat. The cable-car station and a large tourist complex were built between 2013 and 2016. A huge temple complex next to the cable car station was finished in 2015. Many paths and stairs connect the diverse sites and facilities, and many bushes and other flowering plants from elsewhere were planted. The site where *D. leave* was found is one of the few ruderal sites which had been gardened during the years of construction, most probably by construction workers. Remains of plant beds, plantings and an irrigation ditch were still to be found.

**Collection**: All sites around the top of Fansipan mountain (if not inaccessible as a result of the steepness of the terrain or impenetrability of bamboo thickets) were searched intensively for gastropods by four zoologists (I. Dedov, N. Simov, R. Bekchiev and P. Beron) on 21.09.2018.

Eight specimens of *D. laeve* were found close to the temple and not far from the cable-car station on an area of just a few square metres. The slugs were found in the middle of the day (13:00–14:00 hrs), some active on the moist soil and some hidden under stones. The air temperature was approximately 15–20°C, with almost no wind and a cloudy sky. *Deroceras laeve* was found nowhere else, but several other, indigenous species, including semi-slugs (Helicarionidae), were collected elsewhere on Fansipan Dedov et al. (2019), but none together with *D. laeve*.

**Treatment of specimens**: The slugs were killed and fixed on the evening of the collecting day, following the methods in Nitz et al. (2009). They were relaxed in a jar with unchlorinated water and some drops of the surfactant SUPRALAN-UF until fully stretched and dead (after 30–60 min). The dead slugs were cleaned of mucus in a sieve under cold running water. For preservation, 96% ethanol was injected with a syringe into the body cavity through the terminal tip of the sole. The specimens were then covered with ethanol (96%) and left for some hours and finally stored in 75% ethanol. The ethanol was changed at least twice over the following days.

Some specimens were photographed alive before they were killed, using a digital camera Panasonic, Lumix DMC-TZ31.

All individuals are adult (judging by the visible and open genital pore: U. Schneppat, personal observation). Six specimens of the series are kept in the Mollusca collection of the Institute of Biodiversity and Ecosystem Research, Bulgarian Academy of Sciences, Sofia (Collection I. Dedov 40339/B–F and H), two specimens (ex Collection I. Dedov 40339/A and G) are stored in the collection of the Senckenberg Museum of Natural History Görlitz, Germany (p23737, p23738).

*Morphological studies*: Seven specimens, preserved in 75% ethanol, were measured using callipers, a metal ruler or with a calibrated eyepiece. Three specimens were dissected: 4039/B by U. Schneppat and p23737 and p23738 by H. Reise. The general dissection method followed Wiktor (2000). The eighth specimen, 40339/H, was excluded from the measurements reported here, because it was obviously sick or senile: it had shrivelled and deformed considerably during fixation and had a necrotic wound in its right body wall. This specimen is kept in 96% ethanol for potential further DNA work.

*DNA sequencing*: DNA was extracted from foot muscle tissue of specimen 40339/G (= SMNG p23738) following the method of Winnepenninckx et al. (1993). For amplification of the standard bar-coding region of the mitochondrial COI gene, we used the primers LCO1490 and HCO2198 (Folmer et al. 1994). The sequencing, in both directions, was carried out by the Laborzentrum BIK-F (Frankfurt/Main (Germany). For further details see Hutchinson et al. (2020).

## Results

**Figure 2.**
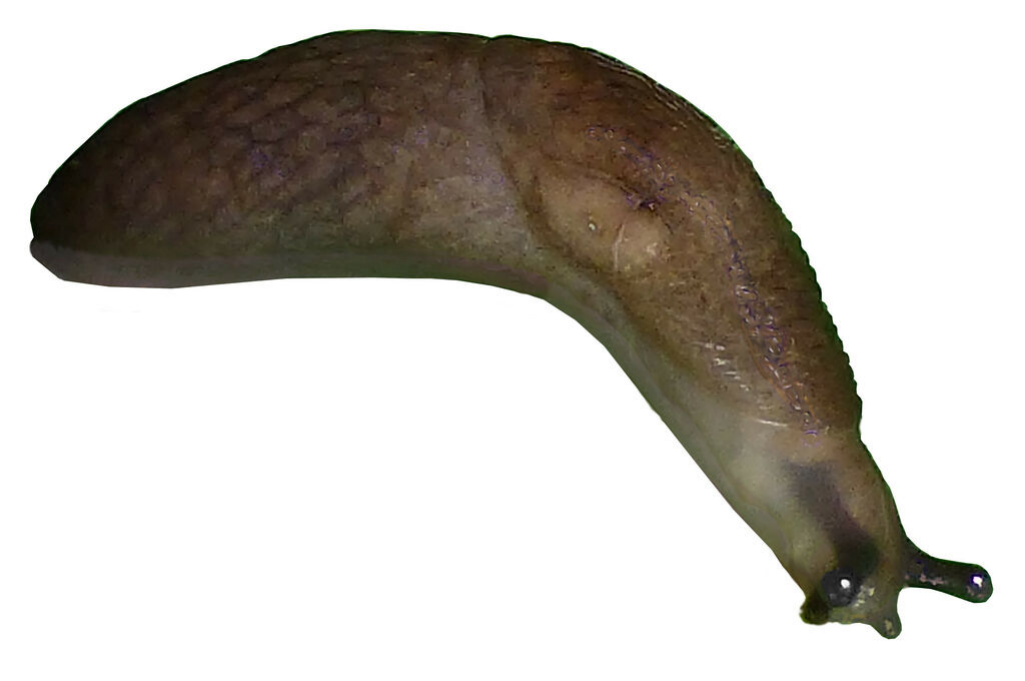
*Deroceras laeve*, Collection I. Dedov no. 40339/A (= SMNG p23737) when alive, 21.09.2018 I. Dedov photo.

Colouration: (*N* = 8) All specimens in the preserved series are of the same general colouration, dark greyish-olive. The coloration of preserved specimens was still largely unchanged after six months in 75% ethanol (Fig. 2)

The ommatophores are entirely black, and the black ommatophorean retractor muscle is well visible through the integument of head and neck. The lower pair of tentacles are uniform pale-grey. The area around the pneumostome is much paler than the mantle and with dark micro-dots along its outer margin. Pigmentation of the sole at first sight appears to be uniform pale grey, but the centre field is slightly lighter. In some specimens, at the outermost end of the lateral sole fields, there is a higher accumulation of dark pigment, and the tip of the sole appears almost black or at least dark-grey. The body mucus of all specimens observed was colourless, wateryand translucent.

Body: (*N* = 7) Animals with very thin and delicate integument. Total body length of preserved specimens 16–18 mm; mantle length 7.0–9.0 mm (43–50% of the total length); keel very inconspicuous and its length highly variable 1.7–5.0 mm. The tail gradually tapers towards the end, with no truncation and not laterally flattened.

**Figure 3.**
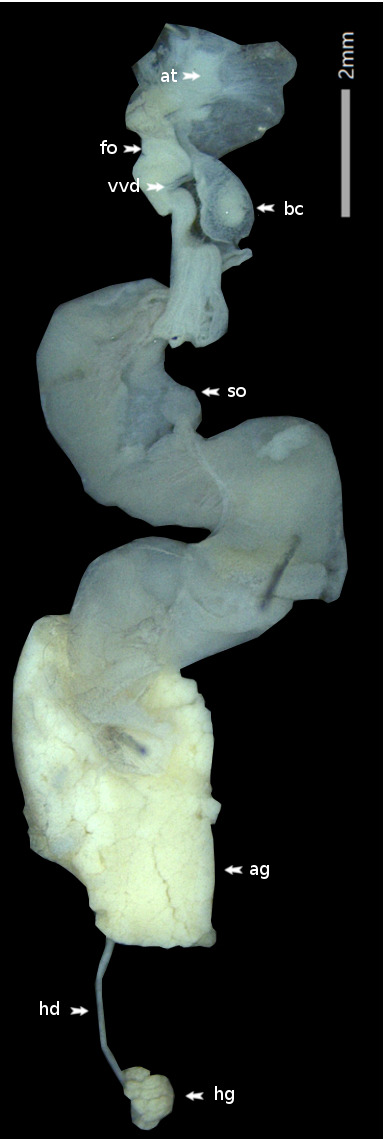
Deroceras laeve, Collection I. Dedov no. 40339/B, genital tract preparation. Abbreviations: ag = albumen gland, at = atrium, bc = bursa copulatrix, fo = free oviduct, hd = hermaphroditic duct, hg = hermaphroditic gland (gonad), so = sperm-oviduct, vvd = vestigial vas deferens. Photograph R. Heim.

Anatomy: (*N* = 3) Penis: completely missing and not even a small vestigial bump found in specimens 40339/A and 40339/B; specimen 40339/G is hemiphallic with a tiny vestigial penis. Musculus retractor penis: completely missing and no remains found at the usual position at the inner body wall. Vas deferens: completely missing in specimen 40339/G, but in specimens 40339/A & B there is a vestigial duct at the proximal end of the free oviduct (Fig. 3). Rectal caecum completely missing.

**Figure 4.**
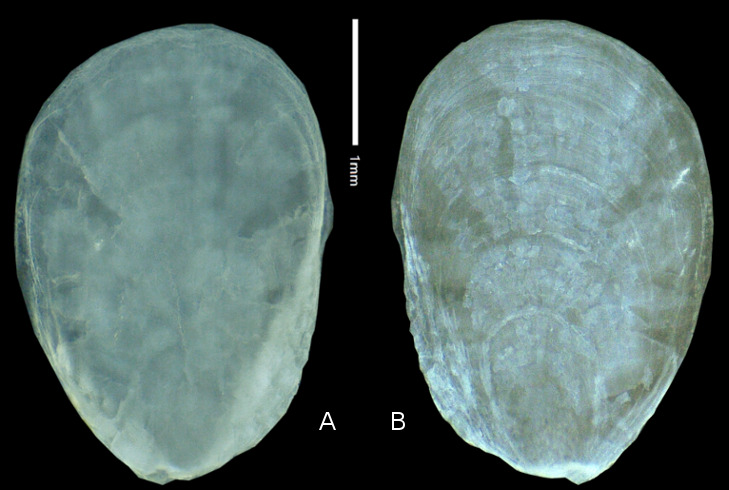
*Deroceras laeve*, Collection I. Dedov no. 40339/B, vestigial shell, ventral (A) and dorsal (B) views. Photograph R. Heim.

Vestigial shell (40339-B): length 3.58 mm, width 2.49 mm. Very flat and straight with almost no cavity ventrally, a little whitish, but almost translucent and without a ventrally thickening calcium-carbonate layer. Periostracum completely colourless, shiny and only visible as a narrow seam at the left and right edge of the shell. Apex almost in the centre of the anterior edge and not very pronounced. Five very regular growth-lines clearly visible all around the shell (Fig. 4)

**Genetics**: We sequenced a 644 bp fragment of the COI gene (GenBank number MT941435). *Deroceras laeve* sequences in GenBank show much variation, but this sequence fully matches one from Massachusetts (GenBank AF239733.1) and also an individual from the Costa Brava, Spain (GenBank number MT941436; collection number SMNG p24032). Another haplotype only one base pair different has been found in Gloucestershire, England (KF894311.1) and Ontario, Canada (MG422202.1). Evidently, the Vietnamese specimen has a widely distributed haplotype, so this does not reliably indicate a likely source population.

## Discussion

The record of a small population of *Deroceras laeve* in northern Vietnam is the first from the Indochinese Peninsula and thereby fills a gap in its range within SE Asia. There are a few other species of this genus which have been spread widely by human activity, such as *D. reticulatum* which has, like *D. laeve*, an almost worldwide distribution (Wiktor 2000, Welter-Schultes 2012, Hutchinson et al. 2014). However, it is more difficult with *D. laeve* to delineate between its natural and introduced ranges. With a supposedly Holarctic natural distribution range (though there is dispute about whether it is native to North America, for example Wiktor (2000), Wiktor and Auffenberg (2002), Welter-Schultes (2012), Gittenberger et al. (2018)), stretching from the arctic Tundra (Berman et al. 2011) to the subtropics, *D. laeve* has by far the largest natural range amongst species of Agriolimacidae (Wiktor 2000). This goes along with an enormous ecological plasticity: it requires some dampness, but can tolerate freezing well below -20 °C, and the short life cycle allows it to grow and reproduce even in extremely short seasons of suitable conditions (Wiktor 2000, Berman et al. 2011). *Deroceras laeve* occurs from sea level to 4800 m and in a variety of forests, wetlands, natural and agricultural habitats (Wiktor 2000, Gittenberger et al. 2018). It is also a frequent pest in greenhouses (Rowson et al. 2014). The species is considered as native in China because the very many records cover most of the country, including the south-eastern regions, and range from lowland to high altitudes (up to 4200 m a.s.l.) and include natural as well as synanthropic habitats (Wiktor 2000). In addition populations along the southern foothills of the Himalayas have been considered as native: several localities in northern Pakistan (Wiktor and Auffenberg 2002, Hlavàč 2004), Nepal (districts of Kathmandu, Taplejung and Panchthar: Bössneck 2006, Budha et al. 2015) and Bhutan (Gaza district in the NW, and further indications from two districts in central and south-eastern Bhutan: Gittenberger et al. 2018).

Populations in the tropics are believed to have been introduced. While *D. laeve* seems widespread in tropical America (e.g. Mexico: Araiza-Gòmez et al. 2017), it is believed to be limited to disturbed habitats in the Asian tropics. The species has been found in the rather temperate and mountainous eastern Indian states of Mizoram and Manipur, about 1000 km east of Fansipan (Surya Rao et al. 2004, Surya Rao et al. 2007), but is also claimed to be rather common in Karnataka, southern India (Jayashankar et al. 2012, NA Aravind personal communication 2020), though based on external characters only and still to be confirmed). It is further known from modified highland habitats of Sri Lanka (Naggs et al. 2003) and Malaysian Borneo (though based on external characters only: Schilthuizen and Liew 2008), from Taiwan (Tsai and Wu 2008), Japan (National Institute for Environmental Studies, Japan 2020; https://www.nies.go.jp/biodiversity/invasive/resources/listen_molluscs.html, accessed 23.08.2020), Papua New Guinea (Wiktor 2003), Queensland, Rarotonga, the Fiji Islands and Hawaii (see Hutchinson et al. 2014 and references cited therein). No *Deroceras* species has been listed for neighbouring Laos (Inkhavilay et al. 2019) or Thailand (Hemmen and Hemmen 2001), nor in the latest list for Vietnam (Schileyko 2011).

The new locality in Northern Vietnam is on a south-eastern outpost of the Himalayan foothills and only about 400 km south of the nearest records in China (Wiktor 2000). Therefore, one might ask whether this is at the furthest margin of its native range. However, there are a number of indications that the population on Fansipan mountain is a recent introduction. 1) It seems to be small and limited to a few square metres; 2) the collecting site is at a highly disturbed site where construction work at a large tourist complex and associated small-scale gardening was finished only a few years before; 3) it is close to the cable-car station. One may assume that some building material for the tourist complex was transported with the newly built cable car and potentially piled up next to the station for some time. *Deroceras* and other terrestrial molluscs are known to cling to imported plants or packages of construction material if stored in the open (Robinson 1999, Chown et al. 2002, Hutchinson et al. 2014). The construction work on the cable car and the tourist complex was mainly undertaken by an Austrian/Swiss company (the Doppelmayr-Garaventa Group, Federal State of Vorarlberg, Austria and Kanton Zug, Switzerland). As they also imported construction material, it seems a realistic option that slugs were imported incidentally from Europe. Provided with at least a certain degree of dampness, *D. laeve* can survive with very little to eat for some time. Its ability to self-fertilise allows also single individuals or even eggs to found a population. The same factors mean that a long-distance introduction from elsewhere than Austria and Switzerland is also plausible. It would require a genetic study involving further loci and many more localities to provide evidence of the likely origin (cf. Hutchinson et al. 2020).

The area of Hoang Lien National Park (29,845 ha in Lao Cai Province, including Sapa town and Fansipan peak at 3143 m a.s.l.) is the site of intersection of two sub-regions of subtropical and tropical high mountains. The year there is divided into two seasons: summer = rainy season from May to October (very wet, with heavy rains) and winter = dry season from November to April. The usual temperature in the summer months is 18–20ºC; in the winter months 10–12ºC. The temperature maximum of the area, measured in April in the lowlands, is 33ºC, the temperature minimum -3ºC. The average annual rainfall is 2,759 mm, with a recorded maximum of 3,484 mm, but rainfall depends on topography and season. About 80–85% of the total annual precipitation falls in summer, mainly June and July, while there is only 50–100 mm rain per month during winter. Humidity is relatively stable with 85–90% during most of the year. It may reach up to 97% and decrease to 65–70% during April. Fog and hoar frost appear often and at all times of the year, especially in winter. On average, there are ca. 160 foggy days per year and six average days with frost (Vietnam Forestry Agency 2013, Nguyen 2017, The meteorological station in Sa Pa 2017). This comparatively mild and wet climate seems very favourable for the humidity-loving *D. laeve*, and it remains to be seen whether the Fansipan population will develop further and expand into surrounding natural habitats.
